# RSW-YOLO: A Vehicle Detection Model for Urban UAV Remote Sensing Images

**DOI:** 10.3390/s25144335

**Published:** 2025-07-11

**Authors:** Hao Wang, Jiapeng Shang, Xinbo Wang, Qingqi Zhang, Xiaoli Wang, Jie Li, Yan Wang

**Affiliations:** Electronic Information Engineering College, Changchun University, Changchun 130022, China; 230402184@mails.ccu.edu.cn (J.S.); wangxb@ccu.edu.cn (X.W.); zhangqq@ccu.edu.cn (Q.Z.); wangxl@ccu.edu.cn (X.W.); lij69@ccu.edu.cn (J.L.); wangy8512@ccu.edu.cn (Y.W.)

**Keywords:** deep learning, remote sensing images, object detection, YOLO

## Abstract

Vehicle detection in remote sensing images faces significant challenges due to small object sizes, scale variation, and cluttered backgrounds. To address these issues, we propose RSW-YOLO, an enhanced detection model built upon the YOLOv8n framework, designed to improve feature extraction and robustness against environmental noise. A Restormer module is incorporated into the backbone to model long-range dependencies via self-attention, enabling better handling of multi-scale features and complex scenes. A dedicated detection head is introduced for small objects, focusing on critical channels while suppressing irrelevant information. Additionally, the original CIoU loss is replaced with WIoU, which dynamically reweights predicted boxes based on their quality, enhancing localization accuracy and stability. Experimental results on the DJCAR dataset show mAP@0.5 and mAP@0.5:0.95 improvements of 5.4% and 6.2%, respectively, and corresponding gains of 4.3% and 2.6% on the VisDrone dataset. These results demonstrate that RSW-YOLO offers a robust and accurate solution for UAV-based vehicle detection, particularly in urban scenes with dense or small targets.

## 1. Introduction

With the continuous development of UAV technologies [1], their application in remote sensing has expanded rapidly across domains such as smart city infrastructure [2], transportation monitoring [3], and ecological surveillance [4]. UAVs are particularly advantageous in urban vehicle detection scenarios due to their ability to capture high-resolution images across extensive areas, which supports applications like traffic density analysis and the detection of unauthorized vehicles. Despite these advantages, performing vehicle detection using UAV imagery still poses several key difficulties. For one, vehicles typically occupy only a small portion of the image frame, often resulting in low visibility and indistinct features, which directly impacts detection precision. Moreover, urban landscapes are visually complex, containing numerous background elements like buildings, trees, and roads that interfere with accurate object identification. Variations in aerial perspectives and inconsistent lighting conditions add further complexity, reducing the reliability of detection results.

At present, deep learning-based vehicle detection methods are generally divided into two main categories based on their processing workflows: two-stage and single-stage detection frameworks. Two-stage models initially use a Region Proposal Network (RPN) to identify potential object regions (ROIs), followed by subsequent classification and localization within these regions. Classic examples of this category include the R-CNN series [5,6,7]. Although such models typically achieve high accuracy, they often involve complex architectures and high computational costs, resulting in slower inference speeds that are less ideal for time-sensitive applications such as real-time traffic monitoring. To overcome these limitations, single-stage detection models have been introduced. These architectures utilize a fully end-to-end convolutional framework that directly predicts both object categories and bounding box coordinates from the input image, omitting the need for a separate proposal stage and thus significantly improving detection speed. Popular single-stage algorithms include YOLO and SSD [8,9]. Owing to their efficiency and low latency, single-stage approaches have gained widespread popularity in real-time vehicle detection applications [10], especially in fields like smart transportation and autonomous driving. Among them, the YOLO series stands out due to its rapid evolution and consistent performance gains in object detection tasks.

In recent years, numerous enhancements have been introduced by researchers to tackle the difficulties associated with vehicle detection in UAV-acquired remote sensing images. For example, Song et al. [11] boosted the model’s capacity to extract fine-grained vehicle features by employing MixUp and Mosaic data augmentation strategies. They further incorporated an Efficient Channel Attention (ECA) mechanism into the backbone to intensify the model’s sensitivity to key feature information. In addition, a decoupled head design was adopted for more effective prediction, which contributed to better detection performance. Huang et al. [12] advanced the DC-SPP-YOLO framework by integrating Dense Connectivity (DC) and Spatial Pyramid Pooling (SPP) modules. This combination greatly enhanced the feature flow within the network, facilitating richer multi-scale representation. Their improved SPP design also allowed for more thorough learning across varied object scales. Similarly, Wang et al. [13] refined YOLOv3 by embedding a spatial pyramid module to improve multi-scale feature extraction capability. Zhu et al. [14] took a comparable approach by embedding both a spatial pyramid and a small-object detection layer into the YOLOv5 architecture, while also introducing the Convolutional Block Attention Module (CBAM) to refine feature attention. These upgrades notably improved the detection performance of small-scale vehicles, especially in cluttered environments. To address problems like missed and false detections in traffic scenarios, Liu et al. [15] proposed the YOLOv8-FDD model. Their approach featured enhancements in three key areas: lightweight detection heads, multi-scale feature refinement, and adaptive upsampling, collectively supporting efficient and accurate real-time detection.

Although the aforementioned studies have contributed to advancements in vehicle detection, several challenges remain: 1. Most existing publicly available vehicle detection datasets feature uniform altitudes and fixed viewing angles and are collected in relatively simple environments, which limits their applicability to real-world scenarios. 2. Robustness is critical in practical applications; however, many current studies focus primarily on ideal or simplified scenes, lacking sufficient adaptability to complex and dynamic environments. 3. Existing object detection algorithms still struggle with accurately identifying small targets, which remains a significant obstacle in high-resolution remote sensing imagery.

To overcome the previously mentioned difficulties, this study introduces an enhanced object detection model named RSW-YOLO, which builds upon the YOLOv8n architecture. The primary objective of this model is to strengthen the network’s capacity for target feature extraction while increasing its resistance to background interference. The proposed approach seeks to maintain computational efficiency while achieving notable gains in detection precision. The main contributions of this work can be summarized as follows:

1. To address the challenges of vehicle detection in urban remote sensing images, we constructed the DJCAR dataset and systematically designed a multi-dimensional data acquisition strategy. The diversity and complexity of the dataset effectively simulate real-world scenarios involving object deformation, occlusion, and illumination variations. This provides a high-quality benchmark for model training and evaluation, bridging the gap in existing datasets for UAV-based urban vehicle detection tasks.
2. To address the difficulty of capturing global contextual relationships between objects and their surroundings—especially in UAV imagery characterized by variable object scales and complex backgrounds—this study incorporates the Restormer module into the YOLOv8n backbone. Leveraging the Multi-Dconv Head Transposed Attention (MDTA) mechanism, the module models long-range feature dependencies that traditional convolutional layers often miss due to their limited receptive fields. In parallel, it integrates the Gated-Dconv Feed-Forward Network (GDFN), which adaptively filters essential feature channels. This combination not only suppresses background noise but also enhances the network’s capability to represent detailed features across multiple scales.
3. Due to the fact that the local features of small targets are easily overwhelmed by background noise, leading to a significant loss of detailed information in high-level feature maps, this paper proposes a small-target detection head. By downscaling the resolution of feature maps and emphasizing crucial channel information, this method effectively minimizes the impact of redundant features, thereby improving the model’s capability to extract fine-grained details of small-scale objects.
4. To overcome the issue of gradient imbalance stemming from the traditional CIoU loss function’s excessive dependence on geometric factors in poor-quality predictions, this work substitutes CIoU with the WIoUv3 loss function. This change helps reduce the overfitting effect caused by low-quality bounding boxes and enhances the influence of high-quality predictions during training. Additionally, WIoUv3 introduces a dynamic, non-monotonic focusing strategy that adaptively modifies the loss weights based on prediction quality, thereby boosting the model’s overall detection effectiveness.
5. This study rigorously verifies the proposed model’s effectiveness in detecting vehicles within remote sensing imagery through ablation studies and comparative performance evaluations. Experimental results on the DJCAR dataset reveal an improvement of 5.4% in mAP@0.5 and 6.2% in mAP@0.5:0.95. Similarly, on the VisDrone dataset, the model achieves gains of 4.3% and 2.6% for mAP@0.5 and mAP@0.5:0.95, respectively. These findings demonstrate that the proposed algorithm delivers a highly accurate solution for UAV-based vehicle detection tasks, effectively lowering the missed detection rate for small targets and enhancing robustness in complex urban environments, particularly related to the following topics: Religions, Risks, Social Sciences, Tourism and Hospitality, and Youth.

## 2. Related Work

### 2.1. Small-Object Detection

Small-object detection has long been recognized as a significant challenge in the field of computer vision, primarily due to the limited pixel information, low contrast, and high sensitivity to background noise associated with small targets. To address these issues, a variety of strategies have been proposed in recent years. One widely adopted approach is multi-scale feature fusion, which aims to integrate feature maps from different layers of a neural network to balance high-level semantic information with low-level spatial details. For instance, Tan et al. [16] introduced the Bidirectional Feature Pyramid Network (BiFPN), which employs a learnable weighting mechanism to effectively fuse low-level detail features with high-level semantic representations. This architecture adaptively enhances feature layers that are more sensitive to small objects, thereby improving detection performance in complex scenes. Another effective strategy involves incorporating contextual information to strengthen the semantic association between small objects and their surrounding regions, thus compensating for weak visual cues. For example, Zheng et al. [17] proposed a framework consisting of three complementary modules—EFEM, SCGBiFPN, and LAPM—each targeting a distinct aspect of contextual utilization in small-object detection, including local multi-scale information extraction, spatial-semantic fusion, and localized attention with noise suppression. Data augmentation has also proven beneficial by increasing the proportion and diversity of small objects in the training set, thereby mitigating foreground–background imbalance. Bosquet et al. [18] developed a comprehensive data augmentation pipeline for small-object detection that integrates a GAN-based object generator with object segmentation, image inpainting, and image blending techniques to produce high-quality synthetic data.

### 2.2. UAV-Based Object Detection

In recent years, object detection based on unmanned aerial vehicle (UAV) remote sensing imagery has garnered increasing attention owing to its broad range of applications, including traffic monitoring, disaster response, and urban surveillance. Compared with ground-level imagery, UAV-captured images introduce distinct challenges such as small-object scales, cluttered backgrounds, and significant scale variations. To facilitate algorithm development and performance evaluation, several benchmark datasets have been established, including VisDrone, UAVDT, and DOTA.

Numerous detection algorithms have been tailored or newly developed to accommodate the specific characteristics of UAV imagery. For instance, Zhong et al. [19] proposed PS-YOLO, a lightweight and efficient detection framework that incorporates a partial convolution-based backbone, a FasterBIFFPN neck for enhanced multi-scale feature fusion, and a GSCD detection head. PS-YOLO achieves notable improvements in both detection accuracy and inference speed on the VisDrone2019 dataset. Fan et al. [20] introduced LUD-YOLO, a UAV-oriented variant of YOLOv8, which integrates adaptive feature fusion, sparse attention mechanisms, and model pruning techniques to achieve superior accuracy–speed trade-offs across the VisDrone2019 and UAVDT datasets. Furthermore, Carion et al. [21] developed DETR, a transformer-based end-to-end detection framework that formulates object detection as a direct set prediction task. By leveraging a bipartite matching loss and learned object queries, DETR eliminates the need for anchors and non-maximum suppression (NMS), thereby simplifying the detection pipeline while maintaining competitive performance on standard benchmarks.

### 2.3. Summary of Advantages over Existing Methods

Despite significant progress in UAV-based object detection, existing methods still face limitations in handling complex urban scenarios, particularly when detecting small-scale vehicles. Traditional convolutional architectures, such as those used in YOLO or its lightweight variants like PS-YOLO and LUD-YOLO, often struggle to capture long-range contextual dependencies, which are critical for distinguishing small targets from cluttered backgrounds. Although multi-scale fusion and attention mechanisms have been employed to enhance feature representation, they frequently lack explicit modeling of global spatial relationships and fail to suppress redundant or noisy features effectively.

To address the aforementioned limitations, the proposed RSW-YOLO introduces three key innovations to enhance detection performance under complex UAV-based scenarios. First, a Restormer module is integrated into the backbone network to incorporate multi-dimensional self-attention mechanisms. This design enables the model to effectively capture long-range dependencies and intricate spatial relationships that are often missed by conventional convolutional layers, thereby improving robustness in densely structured and scale-variant environments. Second, a dedicated detection head for small objects is designed to emphasize informative channels while suppressing redundant and irrelevant features. Third, the conventional Complete IoU (CIoU) loss function is replaced with the more adaptive WIoUv3, which dynamically adjusts the contribution of each prediction based on its quality. This substitution mitigates gradient imbalance caused by low-quality predictions and strengthens the influence of high-quality samples during training, resulting in more accurate and stable localization under conditions such as occlusion, low contrast, and object deformation.

These enhancements are validated through rigorous experiments on both the DJCAR and VisDrone datasets, where RSW-YOLO consistently outperforms baseline models in terms of both mAP@0.5 and mAP@0.5:0.95. These results demonstrate that the proposed approach provides a more effective and reliable solution for UAV-based vehicle detection, particularly in complex urban environments.

## 3. Materials and Methods

YOLOv8 [22] is an efficient object detection algorithm developed by Ultralytics. Its core architecture consists of three key modules: 1. The backbone adopts an enhanced CSP-Darknet53 structure, which extracts multi-scale features through the C2f and SPPF modules. 2. The neck module adopts a dual-path feature fusion strategy by integrating a top-down Feature Pyramid Network (FPN) with a bottom-up Path Aggregation Network (PAN), effectively improving the model’s ability to represent features across multiple scales. 3. The detection head utilizes a decoupled architecture, where classification and localization are processed independently, leading to enhanced prediction accuracy. This structural design contributes to better performance in detecting small objects and strengthens robustness in complex environments, all while maintaining the low latency and high-speed inference typical of the YOLO family. The YOLOv8 series is available in five variants of escalating size and accuracy: YOLOv8n, YOLOv8s, YOLOv8m, YOLOv8l, and YOLOv8x. Among these, YOLOv8n stands out for its compactness and speed, striking an effective balance between computational efficiency and detection precision. As a result, YOLOv8n is adopted as the baseline model in this study. Nonetheless, YOLOv8n still encounters difficulties in detecting small-scale objects and dealing with visually complex backgrounds, particularly in remote sensing scenarios. These shortcomings underscore the necessity for further enhancement in feature learning and noise suppression.

**Figure 1:**
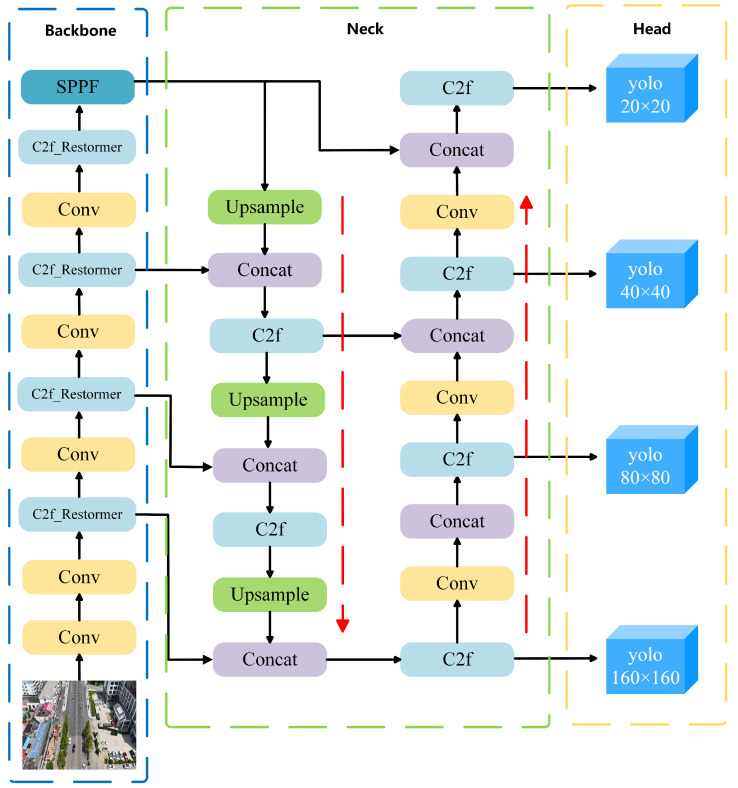
RSW-YOLO network structure diagram.

To enhance detection performance for vehicle targets across varying scales—particularly in the presence of small objects and cluttered backgrounds—this paper introduces an improved model named RSW-YOLO, which is built upon the YOLOv8n architecture. The goal is to address the shortcomings of traditional models under complex visual conditions. The first enhancement involves integrating the Restormer module into the YOLOv8n backbone. By leveraging a self-attention mechanism, this module captures long-range feature dependencies, enabling the model to better adapt to scale diversity and complex scenes while strengthening multi-scale feature fusion. Secondly, the model incorporates a dedicated small-object detection head. This component lowers the resolution of feature maps and focuses on essential channel information, thereby reducing noise from redundant features and improving the precision of small-target localization. Finally, the original CIoU loss is replaced by the more advanced WIoU loss function, which dynamically adjusts weight contributions based on the quality of predicted boxes. This mechanism enhances the model’s stability and accuracy in challenging detection scenarios. The architecture of the proposed RSW-YOLO is visually presented in Figure 1.

### 3.1. Restormer Model

**Figure 2:**
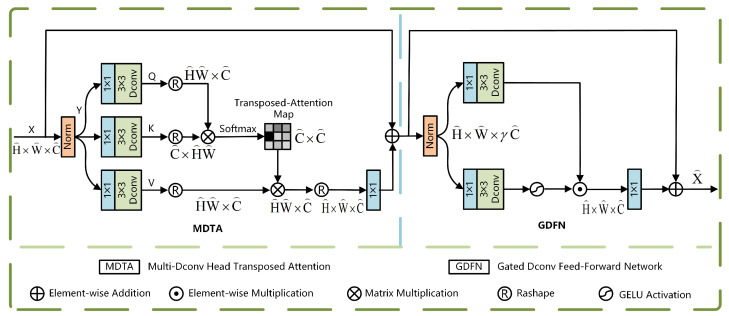
Restormer module.

The Restormer architecture [23] is primarily built upon two essential modules: the Multi-Dconv Head Transposed Attention (MDTA) and the Gated-Dconv Feed-Forward Network (GDFN). Specifically, MDTA adopts a multi-head self-attention architecture, which captures inter-feature relationships in parallel across multiple subspaces, thereby significantly enhancing the expressive capability of feature representations. This self-attention mechanism effectively models long-range dependencies between positions within the feature sequence by computing attention weights between any two positions, enabling global feature interaction. As a subsequent processing unit, GDFN incorporates a gating control mechanism at its core. This architecture facilitates both the regulation of internal information flow and the selective fusion of features, which enhances the network’s capability to detect and capture essential information. The structural layout of the Restormer module is illustrated in Figure 2, where R represents the real-valued space, H×W denotes the spatial dimensions, and *C* stands for the number of feature channels.

**Figure 3:**
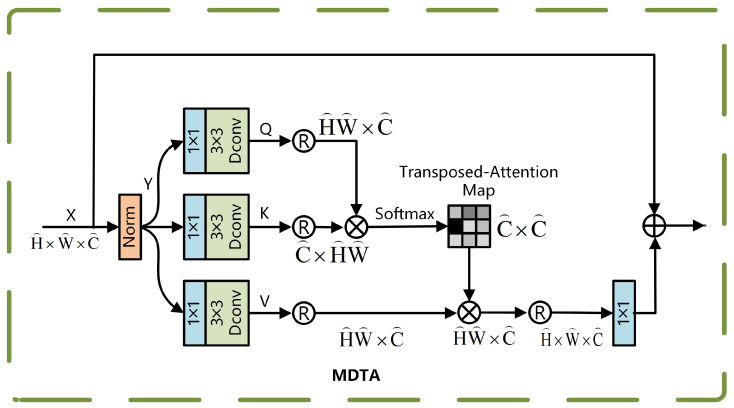
MDTA module.

The MDTA module employs a multi-head attention mechanism. Its working principle involves linearly projecting the input features into multiple parallel subspaces (i.e., heads), where each subspace independently performs attention computations. The outcomes of these attention calculations are subsequently combined and fed into a secondary projection layer to generate the final output. This multi-head parallel processing design enables the model to analyze feature relationships from multiple perspectives, thereby significantly enhancing the diversity of feature representations. The structure of the MDTA module is illustrated in Figure 3.

The process begins as follows: the input feature map $Y\in R^{H\times W\times \hat{C}}$ is first aggregated across channels using a 1×1 convolution $W_{p}$, followed by a 3×3 deep convolution $W_{d}$ to extract spatially localized features, generating the query *Q*, key *K*, and value *V*:

$$Q=W_{d}^{Q}W_{p}^{Q}Y,\;K=W_{d}^{K}W_{p}^{K}Y,\;V=W_{d}^{V}W_{p}^{V}Y.$$

Reshape *Q*, *K*, and *V* into matrix forms:

$$\hat{Q}\in R^{HW\times \hat{C}},\;\hat{K}\in R^{\hat{C}\times HW},\;\mathrm{and}\;\hat{V}\in R^{HW\times \hat{C}},$$

and compute the cross-channel attention weights using the covariance matrix. The corresponding computation is presented in Equation (1):

(1) $$Attention\left(\hat{Q},\hat{K},\hat{V}\right)=\hat{V}\times Softmax\left(\hat{K}\times \hat{Q}/\alpha \right)$$

In this context, α serves as a learnable deflation parameter used to adjust the smoothness of the attention distribution. The channels are divided into *h* heads for parallel computation, and the final multi-head output is fused using a 1×1 convolution $W_{p}$, as computed in Equation (2):

(2) $$\hat{X}=W_{p}\left(\mathrm{Concat}\left(head_{1},head_{2},\ldots ,head_{h}\right)\right)+X$$

*X* and $\hat{X}$ represent the mappings of input and output features. The MDTA mechanism is well-suited for real-time processing of high-resolution images from UAVs, while effectively preserving long-range dependencies through global interaction across channel dimensions.

**Figure 4:**
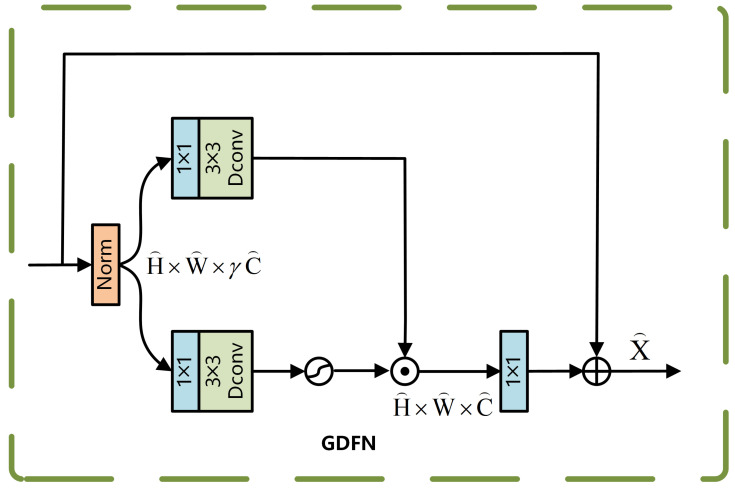
GDFN module.

The GDFN module spatially transforms the input features using gated bilinear feature processing and dynamically adjusts the weights of the transformed features through a sigmoid-activated gating mechanism, enabling selective feature strengthening or attenuation. The structure of the GDFN module is illustrated in Figure 4.

For the normalized feature X, it is processed along two separate paths, after which feature selection is achieved via element-wise multiplication. Equation (3) presents the detailed computation.

(3) $$Gating\left(X\right)=\phi \left(W_{d}^{1}W_{p}^{1}\left(LN\left(X\right)\right)\right)⊙W_{d}^{2}W_{p}^{2}\left(LN\left(X\right)\right)$$

Here, ϕ represents the GELU activation function, and ⊙ denotes element-wise multiplication. The gated result is subsequently combined with the original feature map, and the corresponding computation is presented in Equation (4):

(4) $$\hat{X}=W_{p}^{0}\left(Gating\left(X\right)\right)+X$$

The GDFN suppresses background noise through its gating mechanism, while the deep convolution process enhances local features such as edges, significantly improving the robustness of small-target localization.

**Figure 5:**
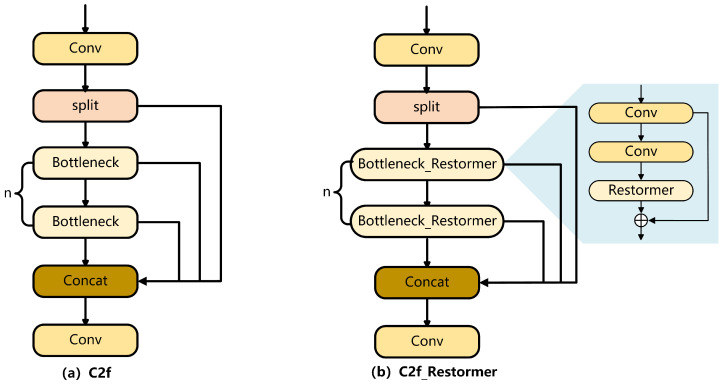
C2f and C2f_Restormer modules.

From an overall architectural perspective, Restormer constructs a multi-level feature learning framework by integrating the MDTA and GDFN units. This layered architecture facilitates the capture of both multi-scale and hierarchical feature representations from input images. In this study, the Restormer module is embedded within the Bottleneck of the C2f component in the backbone network. The structure of C2f after integrating Restormer is shown in Figure 5.

This study incorporates the Restormer module to improve both the precision and robustness of vehicle detection in complex urban environments as seen in UAV imagery. The primary strength of this module lies in its application of multi-head self-attention, which enables the modeling of long-range dependencies and effectively mitigates issues such as object scale variation, background clutter, and frequent missed detections of small targets. In particular, the MDTA mechanism strengthens the model’s capacity to understand contextual relationships between spatially dispersed small objects by facilitating interaction across diverse feature subspaces—for instance, establishing dependencies between occluded vehicles or distant targets of the same class. GDFN incorporates a gating mechanism to dynamically filter key features and suppress redundant background interference (e.g., trees, buildings), thereby improving the precision of object contour localization.

### 3.2. Small-Target Detection Head

**Figure 6:**
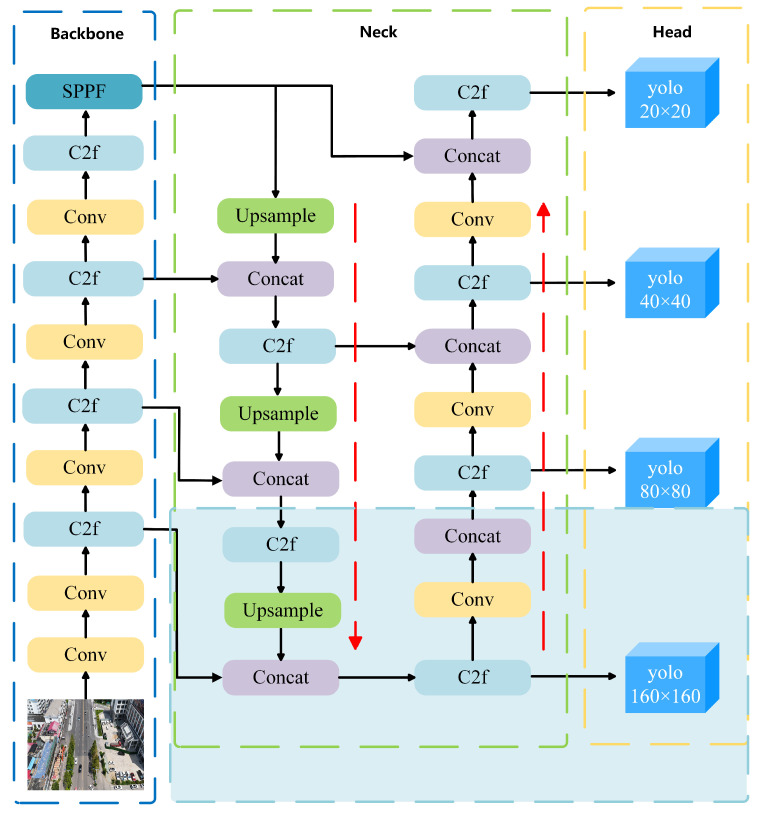
YOLOv8 network architecture after integrating the small-object detection head.

In computer vision tasks, detecting small objects (such as birds, distant vehicles, or fruits on tree leaves) remains a significant challenge. These objects typically occupy a very limited pixel area within an image and are easily overwhelmed by large-scale features in high-level feature maps, leading to a notable decline in detection accuracy. While YOLOv8n performs well in general object detection scenarios due to its efficiency, it tends to be less effective when it comes to identifying small-scale objects. To overcome this limitation, a specialized detection head tailored for small targets is incorporated into the YOLOv8n framework, with the goal of improving its ability to capture fine-grained features, as illustrated in Figure 6.

The fundamental concept of the small-object detection head is to improve the recognition accuracy of small targets by introducing an additional detection branch at the shallow layer of the network. This branch leverages high-resolution feature maps to better retain fine-grained local details. Unlike conventional detection heads, the proposed design focuses on capturing subtle features—such as edges and textures—by operating on more detailed feature representations. It achieves this by compressing the spatial resolution and emphasizing crucial channels, which helps suppress irrelevant information and enhances the precision of small-object localization.

The small-object detection head introduced in this work is essential for UAV-based vehicle detection in urban environments. It addresses the difficulties posed by small target sizes and cluttered backgrounds in remote sensing imagery, leading to improved detection performance for vehicles. Additionally, the detection head effectively minimizes redundant feature interference, enhances the expression of tiny target details (such as edges and textures), and significantly boosts the detection accuracy for small targets.

### 3.3. Loss Function

The traditional CIoU loss function lacks consideration for the balance between difficult and easy samples. As a result, geometric factors—such as aspect ratio and the distance between the centers of predicted and ground-truth boxes—can excessively amplify the negative gradients associated with poor-quality samples. This, in turn, hampers the model’s ability to generalize effectively. To mitigate this problem, the enhanced model adopts the WIoU loss function in place of CIoU. In this framework, let *w* and *h* represent the width and height of the predicted bounding box, while $w^{gt}$ and $h^{gt}$ correspond to the width and height of the ground-truth bounding box. The center coordinates of the predicted and actual boxes are indicated by *b* and $b^{gt}$, respectively, with ρ denoting the Euclidean distance between these centers. The dimensions of the smallest enclosing box that includes both the predicted and ground-truth boxes are given by $w^{c}$ and $h^{c}$. The Intersection over Union (IoU) measures the overlap between the two boxes. Based on these variables, the CIoU loss is defined as in Equations (5)–(7).

(5) $$L_{CIoU}=1-IoU+\frac{\rho^{2}\left(b,b^{gt}\right)}{{\left(w^{2}\right)}^{2}+{\left(h^{2}\right)}^{2}}+\alpha \nu$$

(6) $$\alpha =\frac{\nu }{(1-IoU)+\nu }$$

(7) $$v=\frac{4}{\pi^{2}}{\left(\arctan\frac{w^{gt}}{h^{gt}}-\arctan\frac{w}{h}\right)}^{2}$$

Wise-IoU (WIoU) [24] improves the performance of object detection algorithms by incorporating a dynamic and non-monotonic focus mechanism to assess anchor box quality. Through a gradient modulation technique, it minimizes the influence of detrimental gradients during the training phase, which helps retain high-quality anchor boxes and boosts the model’s robustness. Additionally, WIoU introduces a dual-stage attention mechanism aimed at accelerating convergence, enhancing optimization precision, and strengthening the model’s ability to generalize. Suppose the location (*x*,*y*) within the target box corresponds to ($x^{gt}$,$y^{gt}$); this reflects the loss impact from high-quality anchor boxes. The mathematical expression for WIoU v1 is presented in Equations (8) and (9).

(8) $$L_{WIoUv1}=R_{WIoU}L_{IoU}$$

(9) $$R_{WIOU}=exp\left(\frac{({\left(x-x^{gt}\right)}^{2}-{\left(y-y^{gt}\right)}^{2}}{{\left({\left(w^{c}\right)}^{2}+{\left(h^{c}\right)}^{2}\right)}^{*}}\right)$$

WIoU v3 [25] extends the principles of WIoU v1 by incorporating an adaptive, non-monotonic focus strategy that dynamically modifies loss weightings during training. This dynamic adjustment makes it more suitable for complex object detection tasks, where targets vary in scale, shape, and background context. To improve the robustness and precision of object detection, this study employs WIoU v3 as a substitute for the original loss function. The formulation of WIoU v3 is presented in Equations (10)–(12):

(10) $$L_{WIOUv3}=r\times L_{WIOUv1}$$

(11) $$r=\frac{\beta }{\delta \alpha^{\beta -\delta }}$$

(12) $$\beta =\frac{L_{IOU}^{*}}{L_{IOU}}$$

The loss function is governed by two key hyperparameters: α=1 and δ=3. The parameter α controls the influence of the IoU term in the overall loss formulation, where α=1 ensures a balanced emphasis between the IoU-based localization quality and the sample-wise adaptive penalty. The parameter δ modulates the shape of the reweighting function, controlling the sensitivity of the loss to the deviation between predicted and ground-truth boxes. A larger δ (e.g., δ=3) increases the suppression of low-quality or hard samples, enabling the model to focus on high-confidence, high-IoU predictions. This design helps improve convergence stability and detection precision, particularly in scenarios with dense or ambiguous object layouts.

## 4. Experiments and Analysis

### 4.1. Experimental Setup

The hardware and environment setup used for training is summarized in Table 1.

### 4.2. Dataset

In this study, a vehicle detection dataset tailored for complex urban scenarios, named DJCAR, was constructed. The dataset was collected using a DJI drone (model: DJI Air 3, DJI, Shenzhen, China) to capture vehicle data within urban environments. A multi-dimensional control strategy was employed during data acquisition to enhance sample diversity. Specifically, the drone captured images at varying altitudes, viewing angles, and under different lighting conditions. A total of 944 images were collected, each with a resolution of 4032 × 2268 pixels. The dataset includes 26,075 vehicle instances, among which 25,052 (96.1%) are small objects. Data collection was conducted from April to September 2024. The dataset was split into training, validation, and test sets in a ratio of 8:1:1, containing 755, 94, and 95 images, respectively. The data acquisition settings are detailed in Table 2.

Table 3 illustrates a sample segment from the DJCAR dataset.

To further evaluate the generalization capability of the proposed model, additional experiments are conducted on the public VisDrone dataset [26]—a UAV vision benchmark released by Tianjin University and the Information Technology Laboratory for Data Mining. This dataset is widely adopted for tasks such as object detection and tracking in UAV-based scenarios. VisDrone encompasses a wide range of complex urban environments, including city roads, transportation hubs, campuses, and public squares, and features diverse conditions such as varying illumination and weather. The dataset consists of 10 object categories, including vehicles, pedestrians, and bicycles, with small targets (i.e., objects occupying fewer than 32 × 32 pixels) accounting for approximately 59% of the labeled instances. Each annotation includes detailed attributes such as bounding boxes, occlusion levels, and truncation status, effectively reflecting real-world challenges such as scale variation, dense target distribution, and background clutter in UAV imagery. Compared with traditional object detection benchmarks (e.g., COCO, PASCAL VOC), VisDrone places greater emphasis on the realism and complexity of low-altitude UAV imaging, making it especially suitable for testing algorithm robustness in dynamic and unconstrained environments. The VisDrone dataset contains 8629 images (6471 for training, 548 for validation, and 1610 for testing). The same detection and evaluation strategies were applied to both the DJCAR and VisDrone datasets. For a clearer context of the performance comparison shown in Figure 6, the number of instances per class in VisDrone is as follows: pedestrian (109,187), people (38,560), bicycle (13,069), car (187,005), van (32,702), truck (16,284), tricycle (6387), awning-tricycle (4377), bus (9117), and motor (40,378).

As shown in Table 4, the dataset structure diagrams of both the DJCAR and VisDrone datasets generated during the Ultralytics training process are presented.

Compared with the VisDrone and UAVDT datasets, the proposed DJCAR dataset presents several notable advantages: Higher image resolution: DJCAR contains 944 high-resolution UAV images with a resolution of 4032 × 2268 pixels, which is significantly higher than that of VisDrone (2000 × 1500) and UAVDT (1080 × 540). This allows for better preservation of object details, especially for small or distant vehicles. Diverse flight altitudes: The data were collected from four different altitudes (80 m, 90 m, 100 m, and 110 m), ensuring coverage across low-, mid-, and high-altitude perspectives. This helps to balance object scale (in pixel dimensions) and scene coverage, which is not explicitly controlled in VisDrone. Vehicle-focused annotation: DJCAR specifically targets vehicle detection, with 26,075 annotated vehicle instances. The dataset focuses on urban and suburban road scenes, making it highly relevant for intelligent transportation systems and traffic violation detection tasks. Scene diversity: DJCAR includes scenarios with varying traffic densities, road types, and environmental conditions. Compared to the relatively cluttered and object-diverse nature of VisDrone, DJCAR provides cleaner, vehicle-centric scenes that are more suitable for fine-grained behavior analysis.

The novelty of the DJCAR dataset lies in its high-resolution images, controlled flight altitudes, and vehicle-focused annotations, all of which make it a specialized dataset for vehicle detection. However, we acknowledge that the dataset primarily focuses on urban and suburban road scenes and does not fully represent rural or non-road environments. We recognize that future versions of the dataset could include additional environments to broaden its applicability.

### 4.3. Comparative Experiments

To assess the detection capability of the proposed enhanced model, several widely used evaluation metrics are utilized, such as Precision, Recall, F1-score, mAP@0.5, and mAP@0.5:0.95. The definitions of the relevant variables used in the calculation of these metrics are as follows: True Positives (TPs) refer to positive instances that are correctly identified by the model, whereas False Positives (FPs) correspond to incorrect predictions where negative samples are classified as positive. False Negatives (FNs) are actual positive cases that the model fails to detect, misclassifying them as negative. Additionally, the Intersection over Union (IoU) metric evaluates the overlap between predicted and ground-truth bounding boxes, calculated as the ratio of the intersection area to the total area covered by both boxes.

Precision measures the proportion of true positive predictions among all instances identified as positive by the model. It is calculated using the formula shown in Equation (13):

(13) $$\mathrm{Precision}=\frac{TP}{TP+FP}$$

Recall represents the proportion of true positive predictions relative to the total number of actual positive samples. It is calculated using the formula shown in Equation (14):

(14) $$\mathrm{Recall}=\frac{TP}{TP+FN}$$

The F1-Score balances Precision and Recall, with higher values indicating better performance. It is computed using the formula presented in Equation (15):

(15) $$F1-Score=2\frac{Precision\times Racall}{Precision+Racall}=\frac{2TP}{2TP+FP+FN}$$

Average Precision (AP) is the area under the precision–recall curve. It is calculated using the formula shown in Equation (16):

(16) $$AP=\int_{0}^{1}\mathrm{Precision}\left(Recall\right)dReacll$$

Mean Average Precision (mAP) is the weighted average of the Average Precision (AP) values across all sample categories and is used to evaluate the detection performance of the model across all categories. It is calculated using the formula shown in Equation (17):

(17) $$mAP=\frac{1}{n}\sum_{i=1}^{n}AP_{i}$$

In Equation (17), $AP_{i}$ represents the Average Precision (AP) for the class indexed by *i*, while *N* refers to the total number of classes in the training dataset. mAP@0.5 corresponds to the mean Average Precision with the Intersection over Union (IoU) threshold set to 0.5. On the other hand, mAP@0.5:0.95 represents the mean Average Precision calculated over IoU thresholds ranging from 0.5 to 0.95, with a step size of 0.05. To demonstrate the superiority of the proposed algorithm over other mainstream vehicle detection methods, a comparative evaluation was conducted, several versions of the YOLO series, including YOLOv3-tiny [27], YOLOv5n [28], YOLOv7-tiny [29], YOLOv9t [30], YOLOv10n [31], and YOLOv11n [32], were selected for model comparison experiments. These models represent a range of YOLO architectures, from earlier versions to the latest iterations, and are widely used in target detection tasks. Table 5 and Table 6 present a summary of the experimental outcomes, showcasing the performance of each model on the DJCAR and VisDrone datasets. The primary evaluation indicators include Precision, Recall, mAP@0.5, mAP@0.5:0.95, and the F1-Score.

On the DJCAR dataset, the proposed RSW-YOLO model exhibits clear superiority in UAV vehicle detection tasks. Specifically, it achieves mAP@0.5 and mAP@0.5:0.95 scores of 92.6% and 59.6%, respectively—representing improvements of 5.4% and 6.2% over the baseline YOLOv8n model (87.2% and 53.4%) and outperforming other contemporary detection frameworks. Moreover, the enhanced model records a Precision of 91.2% and a Recall of 85.5%, both surpassing the original YOLOv8n’s performance (88.5% and 79.5%), indicating superior target coverage with fewer false positives. Compared to other mainstream algorithms, the proposed approach maintains a more optimal balance between detection accuracy and Recall. In addition, the F1-Score reaches 88.3%, a 4.5% increase over YOLOv8n, signifying a more refined trade-off between Precision and Recall while effectively suppressing both false alarms and missed detections.

**Figure 7:**
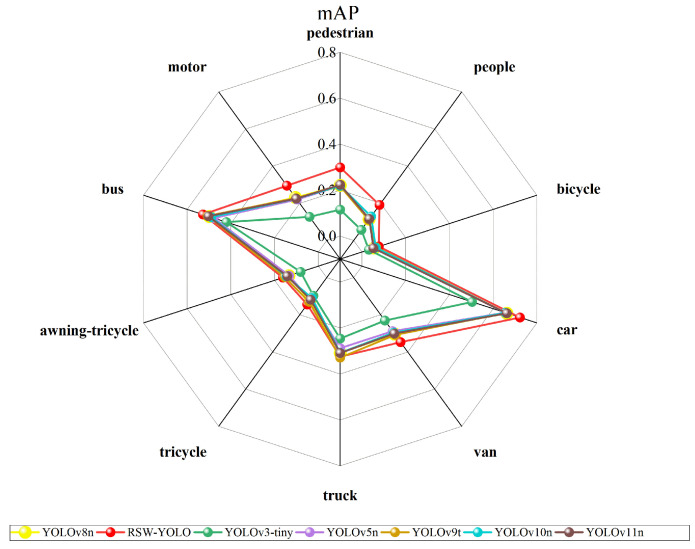
Comparison of other models and RSW-YOLO’s 10 categories.

To further verify its generalization capability, the model was evaluated on the VisDrone dataset, which involves multi-category object detection. On this benchmark, RSW-YOLO also achieved leading results, with mAP@0.5 and mAP@0.5:0.95 reaching 30.9% and 17.6%, respectively. These values exceed those of YOLOv8n (26.6% and 15.0%) by 4.3% and 2.6% and also outperform other leading models. The precision and recall achieved by the proposed model are 41.4% and 33.4%, significantly better than YOLOv8n’s 37.8% and 29.3%. This indicates that the improved model more effectively captures relevant targets while reducing incorrect predictions. Furthermore, the F1-Score of 36.9% represents a 4% improvement over YOLOv8n, underscoring a stronger equilibrium between detection precision and recall. A comparison across object categories is illustrated in Figure 7.

An evaluation of the RSW-YOLO algorithm’s detection capability across ten object categories from the VisDrone dataset was conducted by computing the mean Average Precision (mAP) for each class. The corresponding results are presented in Figure 7. As illustrated, RSW-YOLO demonstrates excellent performance across all 10 categories, with four classes (car, pedestrian, people, and motor) achieving significantly higher mAP@0.5 than the average level of the VisDrone dataset. Notably, the detection of vehicle targets is particularly outstanding, which aligns with the core objective of vehicle detection in urban UAV remote sensing imagery. These findings further affirm the model’s robustness and generalization ability in complex environments.

To evaluate the real-time performance of the proposed detection method, the number of parameters, GFLOPs, and inference speed (FPS) of both the proposed model and the baseline YOLOv8n were measured on an NVIDIA RTX 3090 GPU. The results are summarized in Table 7.

As shown in Table 7, on the DJCAR dataset composed of 4K resolution UAV images, the improved model achieved 60.13 FPS, 11.86 million parameters, and 38.3 GFLOPs, while YOLOv8n reached 110.47 FPS, 3.01 million parameters, and 8.1 GFLOPs. On the VisDrone dataset, which contains images with lower resolutions, the improved model achieved 174.51 FPS, also demonstrating efficient inference performance.

Although the improved model incurs higher computational costs compared to YOLOv8n, it maintains real-time performance even with high-resolution inputs and significantly enhances detection accuracy. These results indicate that the proposed model achieves a favorable trade-off between accuracy and efficiency, making it well-suited for real-time UAV-based object detection applications.

To visually evaluate the impact of the proposed optimizations, a per-sample analysis was carried out on the DJCAR test dataset, focusing on representative samples with high background complexity for visual comparison experiments. The selected samples exhibit challenging characteristics such as significant scale variations within the scene, dense target distributions, and high similarity between targets and background features. These samples effectively reflect the algorithm’s practical detection performance in complex environments. An example of the comparative results is presented in Table 8.

As shown in Table 8, the first column presents the detection results of the baseline YOLOv8 model, while the second and third columns display the outputs of RT-DETR and the proposed RSW-YOLO model, respectively. The selected remote sensing images reflect varying flight altitudes, illumination conditions, and object densities. A quantitative comparison of the detection results is provided in Table 9.

As shown in Table 9, both the YOLOv8n and RT-DETR algorithms exhibit noticeable missed and false detections in complex scenarios involving small vehicle targets, particularly under densely populated conditions. In contrast, the proposed RSW-YOLO model demonstrates significantly enhanced robustness in such challenging environments. It is more effective in detecting small-scale vehicles and substantially reduces both missed and false detection rates. These findings indicate that the proposed method maintains strong detection performance even under complex conditions, effectively mitigating the accuracy degradation typically caused by small object sizes in remote sensing imagery. The improvements clearly highlight the proposed model’s superiority in addressing small-object detection challenges.

### 4.4. Ablation Study

To verify that the improvements proposed in this paper contribute to the enhancement of the algorithm’s accuracy, ablation experiments were conducted on the improved algorithm using the DJCAR dataset. The results of these ablation experiments are shown in Table 10, where “✓” indicates the inclusion of a specific module.

As shown in Table 10, the integration of the Restormer module, the small-target detection head, and the WIoU loss function notably enhances the UAV vehicle detection performance of the improved YOLOv8n model. Specifically, the Restormer module leverages a self-attention mechanism to strengthen multi-scale feature fusion, resulting in a 1.1% increase in mAP@0.5. The customized detection head is designed to improve the recognition of small-scale targets by reducing the resolution of feature maps and emphasizing critical channels, yielding a 4.2% boost in mAP@0.5. Meanwhile, the WIoU loss function mitigates the influence of low-quality predictions through a dynamic weighting strategy, contributing an additional 1.3% gain. When combined, these enhancements produce a clear synergistic effect: mAP@0.5 reaches 92.6%, representing a 5.4% improvement over the baseline YOLOv8n. Moreover, mAP@0.5:0.95 rises to 59.6%, while Precision and Recall improve to 91.2% and 85.5%, respectively. The F1-Score also achieves its peak value, reflecting a well-balanced performance between Precision and Recall. These outcomes confirm the complementary benefits of the proposed components and highlight their effectiveness in handling complex scenarios and detecting small-scale objects.

## 5. Discussion

The experimental results demonstrate that the proposed RSW-YOLO model achieves significant improvements in detection performance on both the DJCAR and VisDrone datasets, particularly excelling in small vehicle detection under complex UAV scenarios. Compared with the baseline model YOLOv8n, RSW-YOLO achieves an increase of 5.4% in mAP@0.5 and 6.2% in mAP@0.5:0.95 on the DJCAR dataset, while achieving 4.3% and 2.6% gains, respectively, on the VisDrone dataset. These improvements validate the effectiveness of the proposed designs, including long-range feature modeling via the Restormer module, a dedicated detection head for small objects, and the use of the WIoU loss function to enhance localization robustness.

However, the performance improvements achieved by RSW-YOLO are accompanied by a noticeable increase in computational complexity. Specifically, the model comprises 11.86 million parameters and incurs a computational cost of 38.3 GFLOPs on the DJCAR dataset, which is substantially higher than that of the baseline YOLOv8n. Despite maintaining real-time processing capability—with an inference speed of 60.13 FPS on 4K UAV imagery—the elevated computational requirements may hinder its deployment on resource-limited UAV platforms. In comparison, ultra-lightweight detectors such as YOLOv5n and NanoDet [33] provide faster inference and lower power consumption, albeit with a reduction in detection accuracy. This comparison underscores the inherent trade-off between model accuracy and computational efficiency in UAV-based applications.

It is also important to note that the inference speed of the RSW-YOLO model exhibits strong resolution dependency. Although the model achieves over 60 FPS on the 4K resolution DJCAR dataset and even higher speeds on lower-resolution datasets such as VisDrone, its actual performance may vary significantly depending on image resolution. In the context of this study, the term “eal time” refers to an overall processing speed—including preprocessing, model inference, and postprocessing—exceeding 30 FPS, which is generally sufficient to meet the requirements of UAV-based vehicle detection tasks. Therefore, while the proposed model demonstrates real-time capability across both high- and low-resolution scenarios, its practical deployment should take into account the resolution of input images and the computational constraints of the target platform.

Another limitation arises in scenarios involving dynamic motion blur, particularly under low-light conditions with fast-moving vehicles. While the Restormer backbone partially mitigates these issues, the model still faces challenges in such settings. The integration of additional attention mechanisms or object-relation modeling techniques may further enhance robustness in these complex environments.

In summary, RSW-YOLO achieves a promising balance between accuracy and inference speed and demonstrates strong potential for high-resolution UAV-based detection tasks. Nevertheless, future work should focus on reducing model complexity through pruning, quantization, and other compression strategies. Additionally, expanding the diversity of training datasets will be essential for enhancing real-world deployment capability and further improving model generalizability.

## 6. Conclusions

In this study, we proposed RSW-YOLO, an enhanced UAV-based vehicle detection framework designed to address key challenges in remote sensing imagery, including small-object detection, scale variation, and background clutter. Built upon the YOLOv8n architecture, RSW-YOLO integrates a Restormer module to capture long-range dependencies and global spatial context, incorporates a dedicated detection head to emphasize fine-grained features of small-scale vehicles, and adopts a reweighted IoU loss function (WIoUv3) to improve localization robustness. These components work synergistically to enhance the extraction of informative features while effectively suppressing irrelevant background noise.

To further support research in UAV-based vehicle detection, we constructed the DJCAR dataset, which contains high-resolution urban road scene images captured at multiple flight altitudes. This dataset offers a comprehensive benchmark for evaluating detection performance under realistic and complex aerial scenarios.

Extensive experiments conducted on both the DJCAR and VisDrone datasets demonstrate the effectiveness of the proposed method. Specifically, RSW-YOLO achieves improvements of 5.4% and 6.2% in mAP@0.5 and mAP@0.5:0.95 on the DJCAR dataset, and 4.3% and 2.6% on the VisDrone dataset, respectively. These results validate the robustness and generalization capability of RSW-YOLO across diverse UAV imaging conditions.

In future work, we plan to further enhance the computational efficiency of the model and conduct cross-dataset validation to comprehensively evaluate its transferability. In addition, we aim to expand the DJCAR dataset by introducing more diverse lighting conditions, such as nighttime and adverse weather scenarios, to improve its applicability in real-world intelligent transportation systems.

## Figures and Tables

**Table 1:** Hardware platform and environmental parameters.

Parameter	Configuration
CPU	14 vCPU Intel(R) Xeon(R) Gold 6330
RAM	24 GB
GPU	NVIDIA GeForce RTX 3090
CUDA Version	11.8
Python Version	3.11
PyTorch Version	2.2.0
epoch	200
batch-size	8
optimizer	SGD

**Table 2:** The information comparison of four datasets.

	Range/Interval	Description
Flight Height	80 m, 90 m, 100 m, 110 m (10 m intervals)	Covers low-, medium-, and high-altitude viewpoints to balance target resolution and scene coverage
Camera Angle	$-40^{\circ }$, $-50^{\circ }$, $-60^{\circ }$, $-70^{\circ }$, $-80^{\circ }$ ($10^{\circ }$ intervals)	Simulates target deformation and occlusion under different observation angles from tilt to nadir
Shooting Time	08:00–10:00 (morning), 12:00–14:00 (midday), 16:00–18:00 (evening)	Captures scenes under varying illumination (sunny, cloudy), color temperatures, and low-light conditions.

**Table 3:** Examples from the DJCAR dataset.

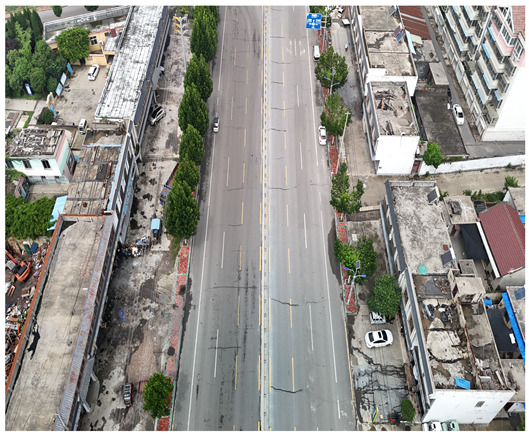	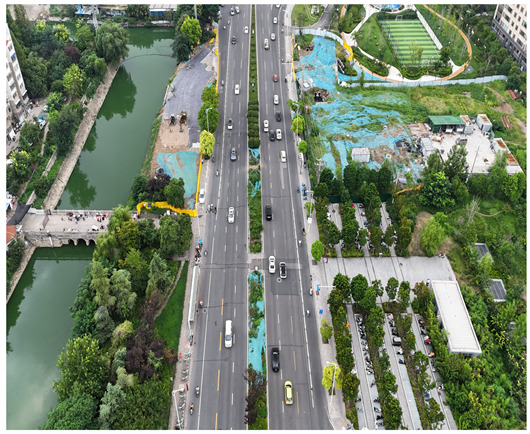	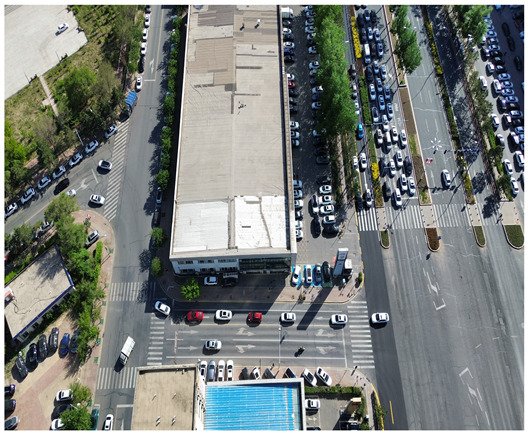
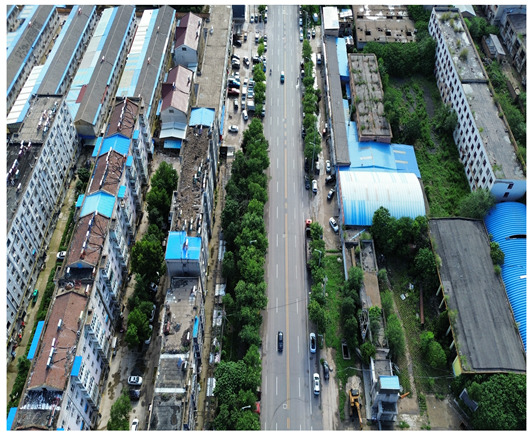	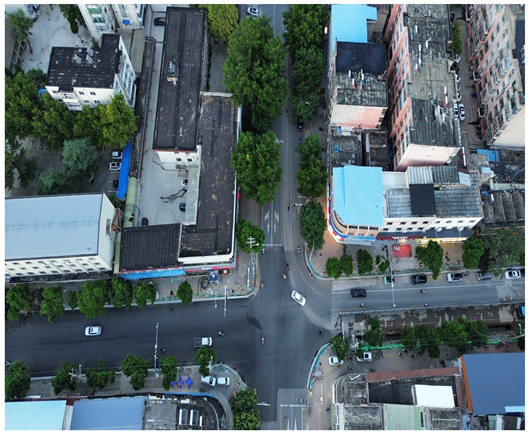	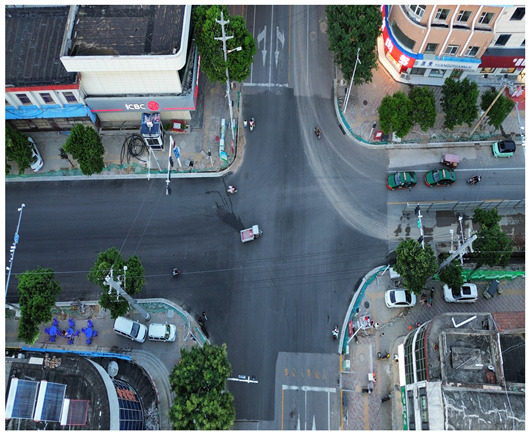

**Table 4:** Structure diagrams of the DJCAR and VisDrone datasets.

DJCAR	VisDrone
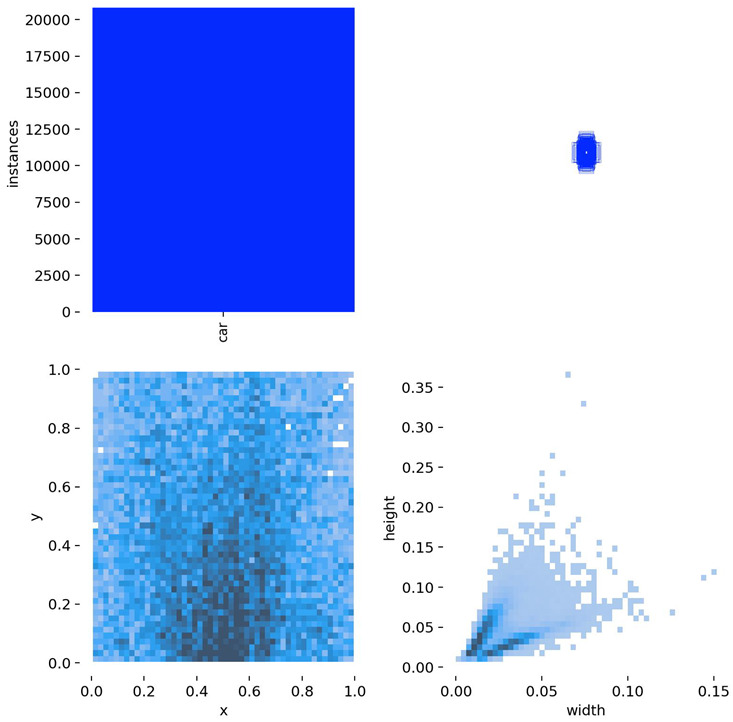	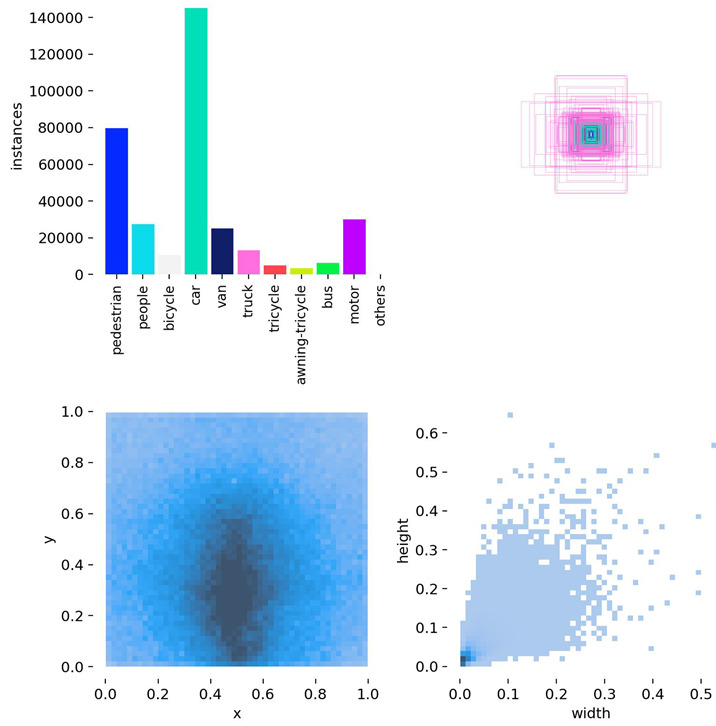

**Table 5:** Results of comparison experiments on DJCAR dataset.

Algorithms	P/%	R/%	F1-Score	mAP@0.5	mAP@0.5:0.95
RT-DETR	80.0	72.9	76.3	81.0	46.6
YOLOv3-tiny	80.2	59.2	68.2	68.2	39.6
YOLOv5n	87.7	81.7	84.6	87.7	53.6
YOLOv8n	88.5	79.5	83.8	87.2	53.4
YOLOv7-tiny	84.5	79.4	81.9	86.5	46.8
YOLOv9t	88.7	78.9	83.6	87.2	53.4
YOLOv10n	84.5	78.6	81.5	85.7	52.9
YOLOv11n	87.2	80.7	83.9	87.5	54.0
ours	**91.2**	**85.5**	**88.3**	**92.6**	**59.6**

The bold values indicate the best performance results.

**Table 6:** Results of comparison experiments on VisDrone dataset.

Algorithms	P/%	R/%	F1-Score	mAP@0.5	mAP@0.5:0.95
YOLOv3-tiny	33.7	21.4	26.1	19.1	10.4
YOLOv5n	36.0	28.7	31.9	25.7	14.4
YOLOv8n	37.8	29.3	32.9	26.6	15.0
YOLOv7-tiny	36.3	36.0	36.1	29.1	14.7
YOLOv9t	39.2	29.4	33.5	27.1	15.4
YOLOv10n	37.7	29.0	32.8	26.4	14.8
YOLOv11n	38.5	29.1	33.1	26.5	14.9
ours	**41.4**	**33.4**	**36.9**	**30.9**	**17.6**

The bold values indicate the best performance results.

**Table 7:** Parametric comparison.

Dataset	Algorithms	Parameters	GFLOPS	FPS
DJCAR	YOLOv8n	3,005,843	8.1	110.47
	Ours	11,855,364	38.3	60.13
VisDrone	YOLOv8n	3,007,793	8.1	390.74
	Ours	11,856,684	38.4	174.51

**Table 8:** Detection result comparison of the algorithms.

	YOLOv8n	RT-DETR	Ours
(**a**)	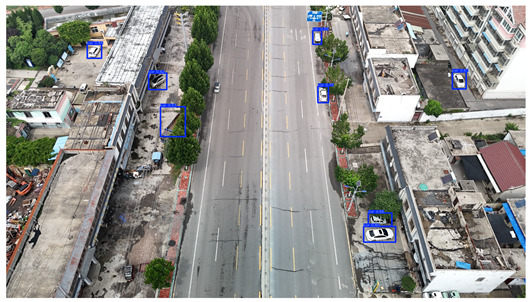	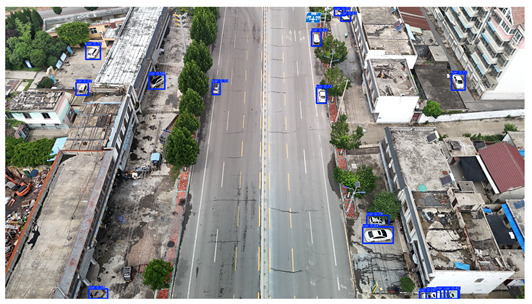	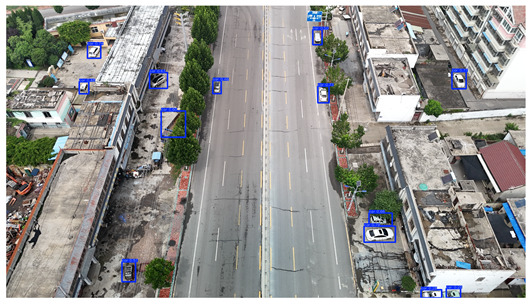
(**b**)	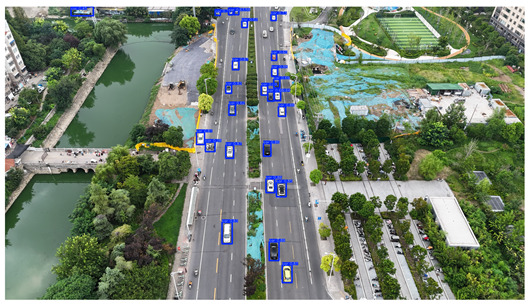	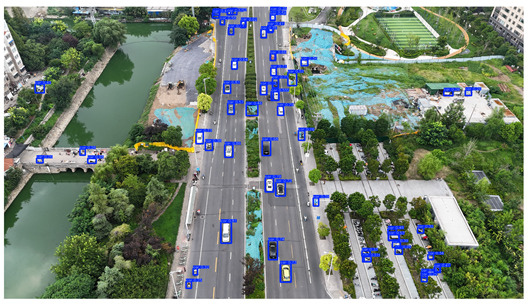	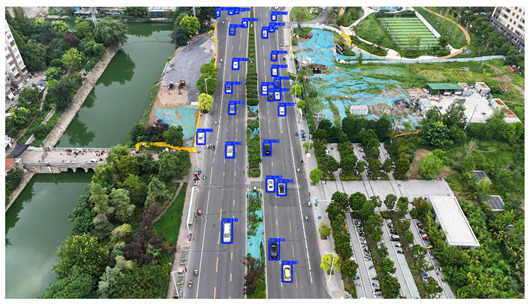
(**c**)	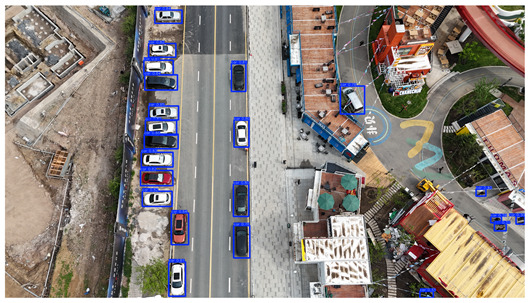	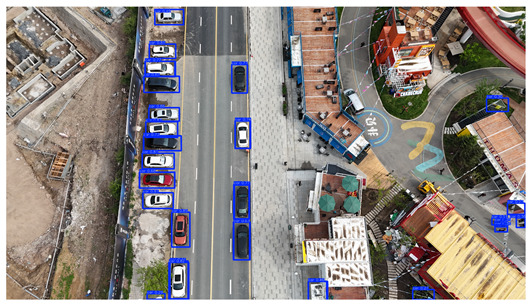	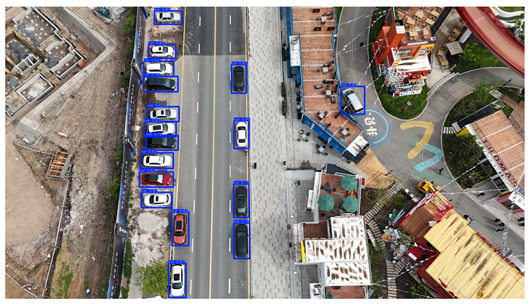
(**d**)	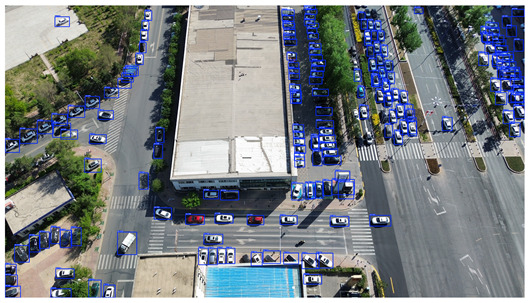	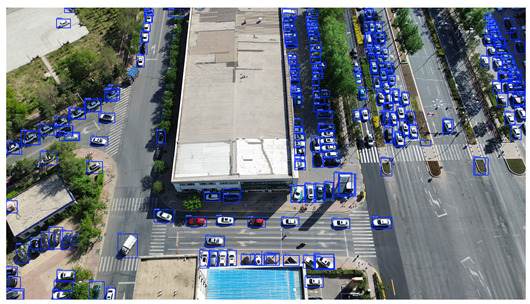	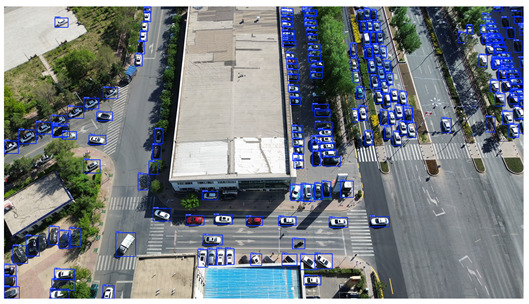
(**e**)	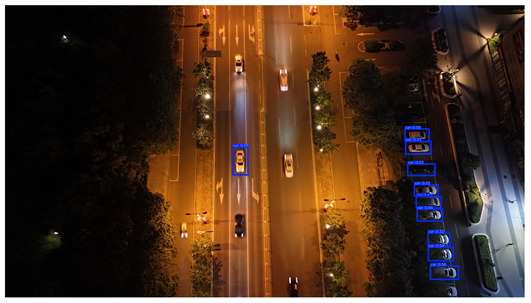	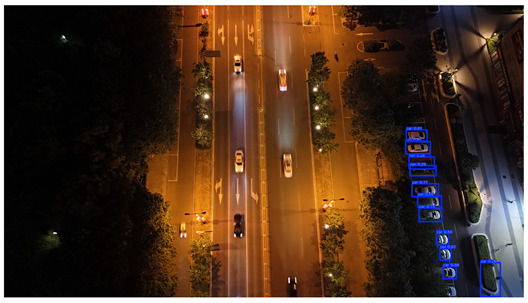	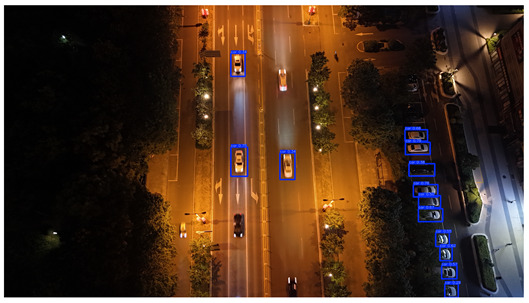

**Table 9:** Detection result statistics of the models on actual images.

Image	Type	YOLOv8n	RT-DETR	Ours
(a)	Wrong	0	3	**0**
	Missed	6	1	**1**
(b)	Wrong	2	23	**0**
	Missed	6	3	**3**
(c)	Wrong	5	9	**0**
	Missed	0	1	**0**
(d)	Wrong	19	50	**15**
	Missed	27	15	**14**
(e)	Wrong	0	2	**0**
	Missed	10	11	**7**

The bold values indicate the best performance results.

**Table 10:** Results of ablation experiments for the proposed approach.

YOLOv8n	Restormer	Small Head	WIoU	P/%	R/%	F1-Score	mAP@0.5	mAP@0.5:0.95
✓				88.5	79.5	83.8	87.2	53.4
✓	✓			89.5	80.7	84.9	88.3	55.3
✓		✓		88.7	84.9	86.8	91.4	58.4
✓			✓	88.6	81.3	84.8	88.5	54.3
✓		✓	✓	89.9	83.3	86.5	91.4	57.4
✓	✓		✓	90.1	80.3	84.9	88.8	55.6
✓	✓	✓		90.3	84.0	87.1	91.8	**58.7**
✓	✓	✓	✓	**91.2**	**85.5**	**88.3**	**92.6**	59.6

The bold values indicate the best performance results.

## Data Availability

Due to privacy and data management policies, the DJCAR dataset used in this study is not publicly available at this time. However, we plan to release the DJCAR dataset in the future. Researchers interested in using the dataset for academic purposes may contact the corresponding author to discuss potential data-sharing agreements.
